# Recent advances in the management and understanding of diabetic retinopathy

**DOI:** 10.12688/f1000research.12662.1

**Published:** 2017-11-29

**Authors:** Matthew Powers, Margaret Greven, Robert Kleinman, Quan Dong Nguyen, Diana Do

**Affiliations:** 1 Byers Eye Institute, Stanford University School of Medicine, 2452 Watson Court, Palo Alto, CA, 94303, USA

**Keywords:** diabetic retinopathy, diagnosis, treatment, screening, optical coherence tomography, laser treatment, vascular endothelial growth factor inhibitors

## Abstract

Despite recent advances in the diagnosis and treatment of diabetic retinopathy, this complication remains a steadfast challenge to patients and physicians. This review summarizes recent progress in the diagnosis and management of diabetic retinopathy, including automated screening, optical coherence tomography, control of systemic risk factors, surgical techniques, laser treatment, and pharmaceutical treatment, including vascular endothelial growth factor inhibitors. Recent advances in pharmaceutical treatments, in particular, hold strong promise of halting and sometimes reversing the disease process. Clinicians nevertheless must remain vigilant in their efforts to diagnose and treat this disease early in its course.

## Introduction

Diabetic retinopathy (DR) is the leading cause of blindness among patients 25–74 years old in industrialized countries. In the US alone, there are 29 million Americans living with diabetes mellitus (DM). The diagnosis, prevention, and treatment of DR thus represent a formidable challenge to the health-care system. Vigilant awareness of the continually evolving landscape of medical knowledge is critical to stemming this crisis. This review aims to provide important clinical updates in the management of DR.

## Update on diagnosis

The cause of DR is multifactorial, and the primary contributor likely is chronic capillary non-perfusion and retinal ischemia. The signaling molecules insulin-like growth factor-1, platelet-derived growth factor, angiopoietin, and most importantly vascular endothelial growth factor (VEGF) all play a role in the subsequent development of microangiopathy ^1^. Recent evidence also suggests that neurodegeneration is an early event in the pathogenesis of DR ^2, 3^. From a clinical standpoint, it is clear that the primary driving factor in this pathogenesis is uncontrolled blood glucose levels, with blood pressure and blood lipid composition also playing important roles. The diagnosis of DR remains clinical in nature. The gold standard for diagnosis is a dilated eye exam and serial fundus photos. Careful attention should be given to fundoscopic features of retinopathy, including microaneurysms, intraretinal hemorrhage, hard exudates, venous beading, intraretinal microvascular anomalies (IRMAs), and, in the proliferative form, signs of neovascularization (NV). Several emerging technologies have demonstrated promise in assisting with the diagnosis of sight-threatening retinopathy. Researchers in collaboration with Google recently created a deep learning artificial neural network trained to detect retinopathy based on fundoscopic images and achieved sensitivity of 97.5–96.1% and specificity of 93.4–93.9% in detecting referable disease. In a hypothetical population with a prevalence of 8%, this translates to impressive positive and negative predictive values of 99.8% and 99.6%, respectively ^4^. Such automated systems hold the potential of offsetting the surge in demand for screening.

## Update on imaging

**Figure 1.**
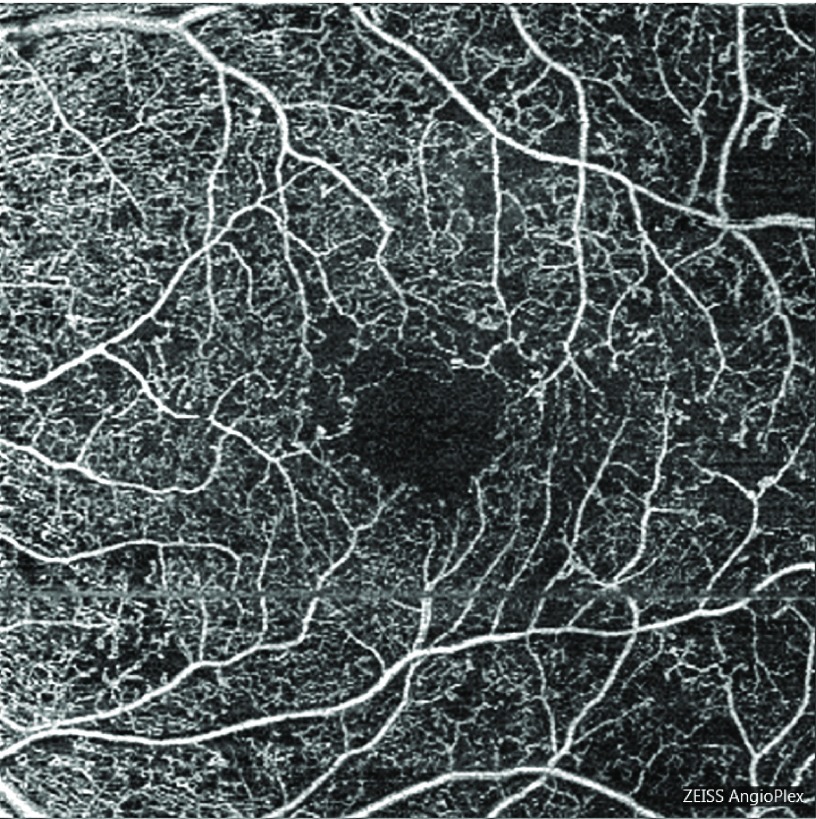
Optical coherence tomography angiography image of a left eye in a patient with severe non-proliferative diabetic retinopathy, displaying enlargement and irregularity of the foveal avascular zone, and temporal capillary dropout. Optical coherence tomography angiography image of a left eye in a patient with severe non-proliferative diabetic retinopathy, displaying enlargement and irregularity of the foveal avascular zone, and temporal capillary dropout.

Imaging modalities, especially optical coherence tomography (OCT) and fluorescein angiography (FA), now play a crucial role in the diagnosis and management of complications of DR, particularly for diabetic macular edema (DME) and subtle NV, respectively. Newer modalities of OCT, including optical coherence tomography angiography (OCTA), hold the promise of further expanding the role of imaging. By using variation in phase and intensity of a light signal to infer vascular structures ^5^, OCTA is able to resolve vascular details not achievable by conventional FA, such as the deep and superficial capillary plexus ^6^. Several authors have argued that OCTA is at least equal to FA in terms of ability to detect macular complications of DM, as it can detect areas of IRMA and NV ^7^. Other microvascular changes seen on OCTA, specifically decreasing capillary density, branching complexity, and increasing average vascular caliber, are all associated with worsening DR ( Figure 1) ^8^. Intriguingly, some forms of retinal edema may be visible on OCTA but will not appear as fluid pockets on OCT or late staining on FA ^9^. Important drawbacks of the technology are the small field of view and relative deficiency in detection of microaneuryms ^9^.

## Update on glycemic control and systemic factors

The Diabetes Control and Complications Trial (DCCT) and United Kingdom Prospective Diabetes Study demonstrated that glycemic control reduces microvascular complications for type 1 and type 2 diabetics, respectively ^10^. A recent 30-year follow-up of the DCCT, and more specifically the Epidemiology of Diabetes Interventions and Complications study, also showed the importance of hemoglobin A1c (HbA1c) control, revealing a 50% risk reduction of retinopathy progression in intensive glycemic control patients, despite a subsequent increase (and corresponding decreasing in standard control patients) to a mean HbA1c value of 8% ^11, 12^. The Diabetic Retinopathy Clinical Research Network (DRCR.net) recently released the results of Protocol M, which showed that the addition of personalized risk assessments and education about glycemic control during ophthalmic office visits did not affect glycemic control ^13^.

The Action to Control Cardiovascular Risk in Diabetes Follow-on (ACCORDION) group also recently released follow-up data of the Action to Control Cardiovascular Risk in Diabetes (ACCORD) trial. Four-year outcomes of patients with type 2 DM were compared between a standard therapy group (goal HbA1c 7.0–7.9%, mean HbA1c 7.7% during the study) and intensive glucose control group (goal HbA1c <6%, mean HbA1c 6.4%). Despite normalization after the study period, only 5.8% of intensive control patients had a three-step Early Treatment Diabetic Retinopathy Study (ETDRS) worsening, compared with 12.7% of standard therapy patients ^14^. This interesting result suggests that there is a form of metabolic memory in diabetic patients, which confers a protective effect of even short periods of intense glycemic control. The ACCORDION group also looked at the effects of blood lipid composition and blood pressure, showing that intensive blood pressure control had no effect on DR progression but that treatment with fenofibrate did show a reduction in progression. The Fenofibrate on the Need for Laser Treatment for Diabetic Retinopathy (FIELD) study echoed these findings, showing that patients taking fenofibrate had a reduced need for laser therapy. Interestingly, non-lipidic mechanisms appear to have a more central role in this beneficial effect than lipidic mechanisms ^15, 16^.

It is important to note, however, that the ACCORD study was halted prematurely when it appeared that all-cause mortality was greater in intensive glycemic control patients. It is apparent that cardiovascular disease was the primary cause of mortality, but the exact reason for this discrepancy remains unclear. This effect was not seen in similar studies, but polypharmacy has been suggested as a possible factor ^17^. Based on the above studies, it is advisable to aim for an HbA1c of less than 7% and to avoid polypharmacy. Importantly though, glycemic goals need to be carefully individualized on the basis of the presence of additional comorbidities, expected longevity, and any other relevant factors.

## Update on surgery and vitreolysis

Vitreoretinal surgery is the standard treatment of several ocular complications of DR. The treatment of choice for non-clearing vitreous hemorrhage remains pars plana vitrectomy. Diabetics can experience macular edema from vitreous traction or epiretinal membranes, both of which are surgically treated. Tractional retinal detachments (TRDs) threatening the macula and combined tractional-rhegmatogenous retinal detachments should be surgically repaired ^18^, although more peripheral TRDs often can be carefully observed ^19^. Diabetic patients with vitreomacular traction (VMT) may also benefit from surgical intervention. The DRCR.net investigated the efficacy of pars plana vitrectomy and membrane peeling for patients with VMT and DME in Protocol D. The study found that nearly 40% of patients had a visual gain of at least 10 letters but that 22% had a worsening of at least 10 letters. This result was concurrent with a host of surgical complications like endophthalmitis (1.1%), vitreous hemorrhage (5.7%), and retinal detachment (3.4%) ^20^. The recent advent of small-gauge vitrectomy may help stem these complications and potentially tip the balance toward earlier surgical intervention.

It has been shown that the presence of a posterior vitreous detachment (PVD) reduces the risk of proliferative diabetic retinopathy (PDR) development, likely because the posterior hyaloid and vitreous act as a scaffold for NV. Ocriplasmin is a proteolytic enzyme agent intended to non-surgically induce a PVD. A trial to investigate its use in patients with diabetes is under way ^21^.

Non-clearing vitreous hemorrhage is a common complication of neovascular disease that is also treated surgically. The original Diabetic Retinopathy Vitrectomy Study showed that early vitrectomy (that is, within six months compared with more than one year) can be useful, especially for type 1 diabetics ^22^. Though the risks and benefits of vitrectomy must be weighed in each case, earlier surgery can be helpful in immediately restoring vision, inducing a PVD, and providing pan-retinal photocoagulation to reduce disease burden ^23^. Anti-VEGF agents have proven useful in the management of vitreous hemorrhage and have been shown to reduce the eventual need for vitrectomy ^24^. Nevertheless, vitrectomy still has a role to play for non-clearing hemorrhage.

Retina surgeons have also incorporated the use of intravitreal VEGF inhibitors when repairing TRDs. In case series, preoperative anti-VEGF injections have been shown to improve outcomes in patients with TRD by regressing active retinal NV and decreasing the likelihood of perioperative bleeding ^25^. However, use of these agents has also been reported to exacerbate TRDs via the ‘crunch’ phenomenon, whereby fibrovascular contraction occurs ^26^, making it more difficult to separate tissue planes ^27^. Newer techniques and devices have the potential to further improve the outcomes in patients with TRDs. Bimanual dissection techniques with chandelier lighting or an illuminated pick can be effective in repair of complex detachments and peeling membranes ^28^. Additionally, mixed-gauge vitrectomy, with 27-gauge instruments inserted through 24-gauge cannulas, can permit access to more of the retinal periphery in these complex cases ^29^.

## Update on laser

The landmark Diabetic Retinopathy Study demonstrated a reduction in severe vision loss in patients with high-risk PDR following prompt treatment with panretinal photocoagulation (PRP) ^30^. The ETDRS originally demonstrated a 50% reduction in moderate vision loss in patients with clinically significant diabetic macular edema (CSME) who underwent immediate focal laser photocoagulation ^31^. Moreover, among patients with PDR or severe non-proliferative diabetic retinopathy (NPDR), combined focal and scatter photocoagulation reduced severe vision loss by 50%. The DRCR.net Protocol K results revealed that among patients with a reduction in CSME 16 weeks after focal/grid laser treatment, 23–63% will continue to improve without further treatment ^32^. PRP remains a mainstay of treatment for proliferative disease. Recently, the results of DRCR.net Protocol S were released, demonstrating that the anti-VEGF agent ranibizumab was non-inferior to PRP for the treatment of PDR. At two years of follow-up, about 53% of patients in the PRP group also received intravitreal ranibizumab for coexisting DME, demonstrating that combination treatment can be helpful in these situations ^33^. How this will affect clinical practice patterns, especially in light of concerns surrounding cost and the inconvenience of regular intravitreal injections, remains to be seen. And while focal laser is still used by many practitioners, anti-VEGF agents have largely displaced it as the primary treatment of DME. There continues to be research into use of focal laser, especially in the form of non-damaging, or subthreshold, laser therapy, although to date this form has shown only marginal benefit compared with conventional laser ^34^. Although many physicians currently use non-damaging laser, there remains to be a clear consensus on its preferred use.

## Update on pharmacologics: ranibizumab

As mentioned previously, anti-VEGF agents are revolutionizing the management of DR. Intravitreal VEGF inhibitors are the first-line agents to treat center-involving DME, and these agents have a growing use in control of proliferative disease as well. Ranibizumab (Lucentis, Genentech, South San Francisco, CA, USA) is the antigen-binding fragment of a humanized murine recombinant monoclonal antibody to VEGF-A ^35^ and is US Food and Drug Administration (FDA)-approved for the treatment of DME and DR at a dose of 0.3 mg monthly.

The Ranibizumab for Diabetic Macular Edema (RIDE and RISE) trials investigated the use of monthly ranibizumab at two doses—0.5 and 0.3 mg—for the treatment of DME ^36^. A secondary analysis of these data also examined the progression of DR in these patients using the diabetic retinopathy severity score (DRSS) ^37^. In patients with NPDR or PDR, there was an at least three-step improvement in 15% and 13.2% of patients using monthly 0.3 or 0.5 mg injections at 36 months, respectively ^37^. Only 3.3% of patients in the sham group achieved this result. The probabilities of progression to PDR in these patients were 39% in the sham group and 18.3% and 17.1% in the 0.3 and 0.5 mg groups, respectively.

Five hundred patients from RIDE and RISE were then offered ranibizumab 0.5 mg as needed, and outcomes at 48 months were examined. At 48 months, 11.2% and 7.6% of patients in the 0.3 and 0.5 mg groups achieved an at least three-step DRSS improvement, respectively. The sham with crossover group, by comparison, saw this improvement in only 4.8%. Additionally, an at least two-step worsening occurred in 2.5% and 11.3% of ranibizumab and sham patients, respectively. Finally, patients originally in the ranibizumab groups had overall lower risk of PDR development than the sham group, a finding which persisted to month 54 ^37^. An additional secondary analysis looked at FA results in the sham group and showed that when these patients were switched to ranibizumab, retinal non-perfusion was halted ^38^. Overall, RIDE and RISE stressed the importance of early and regular therapy with ranibizumab.

The DRCR.net, mentioned above, also examined results of treatment of DME with combinations of laser, steroids, and anti-VEGF in Protocol I. The study was a prospective randomized trial which divided patients with DME into four groups: sham injection plus focal laser (L), triamcinolone injection plus focal laser (T+L), ranibizumab with prompt laser (R+pL), and ranibizumab with deferred laser (R+dL) ^39^. Further analysis then separated these groups into patients with and without PDR at the time of randomization ^40^. For patients without PDR at baseline, worsening of retinopathy at 36 months was reported in 7%, 18%, 23%, and 37% of R+dL, R+pL, L, and T+L groups, respectively. Among patients with PDR at baseline, worsening of retinopathy at 36 months was reported in 18%, 21%, 40%, and 12% among the same respective groups. Additionally, patients receiving injections of any kind (ranibizumab or triamcinolone) had lower rates of vitreous hemorrhage and were less likely to require PRP. The results are strong evidence that ranibizumab can help prevent progression of retinopathy.

As mentioned previously, DRCR.net Protocol S also showed that ranibizumab was non-inferior to PRP in patients with high-risk PDR ^33^. Additional 2016 data showed that patients with PDR treated with ranibizumab showed less progression than patients treated with PRP ^41^. Ranibizumab was recently approved by the FDA for treatment of all forms of DR. It remains to be seen how the results of Protocol S combined with this approval will affect clinical practice patterns.

## Update on pharmacologics: aflibercept

Aflibercept (Eylea, Regeneron, Tarrytown, NY, USA), another widely used agent, is a fusion protein of the human IgG Fc region and the extracellular VEGF receptor ligand binding region, which binds to VEGF-A, VEGF-B, placental growth factor-1 (PlGF-1), and PlGF-2 ^35^. It is FDA-approved for the treatment of DME and DR in patients with DME at a dose of 2 mg every eight weeks after five initial monthly injections. It has been studied recently in the Intravitreal Aflibercept for Diabetic Macular Edema (VISTA and VIVID) trials. In these trials, aflibercept was compared with focal laser for the treatment of DME ^42^. These studies demonstrated the superiority of aflibercept over laser in terms of visual acuity improvement and at least two-step improvement in DRSS. Patients were separated 1:1:1 into groups receiving focal laser, 2 mg of intravitreal aflibercept every four weeks, or 2 mg every eight weeks. Both injection groups initially received five months of monthly aflibercept injections. Patients in either aflibercept group were three times more likely to achieve at least two-step DRSS improvement than patients in the laser group. In both studies, baseline DRSS was correlated with the likelihood of at least two-step DRSS improvement.

A recent publication from the DRCR.net ^43^ evaluated eyes enrolled in Protocol T and reviewed how their DRSS changed after treatment with aflibercept, bevacizumab, or ranibizumab. In this analysis, all three agents were effective in reducing DR. However, in the subset of eyes that had PDR at baseline, aflibercept was more effective in regressing the PDR than bevacizumab or ranibizumab.

## Update on pharmacologics: bevacizumab

Bevacizumab (Avastin, Genentech) is a full-length humanized murine monoclonal antibody that binds to VEGF-A ^35^. It is not FDA-approved for the treatment of DR or DME. The intravitreal bevacizumab or laser therapy in the management of diabetic macular edema (BOLT) study examined the efficacy of bevacizumab versus focal laser for DME. Patients in the bevacizumab group showed significant best-corrected visual acuity (BCVA) improvement over patients in the laser group ^44^. ETDRS retinopathy levels were also included and showed that patients in the bevacizumab arm trended toward DR reduction, although this effect was not statistically significant. This is likely due to the relatively small number of patients enrolled in the study. A recent comparative effectiveness randomized clinical trial conducted by the DRCR.net compared bevacizumab with ranibizumab and aflibercept for DME and found that all three agents are effective treatments, and this was confirmed on two-year follow-up ^45^.

## Anti–vascular endothelial growth factor summary

These and earlier trials of anti-VEGF agents are evidence that early and regular anti-VEGF treatment can halt and sometimes reverse DR. The reason for reversal is still unclear, and elucidation of this mechanism may open the door to new therapies. Given that capillary non-perfusion and retinal ischemia may be the primary contributors to DME and DR progression at baseline, it is possible that halting non-perfusion results in a reversal of the disease in some patients ^37^. Knowing which patients will or will not benefit most from anti-VEGF treatment continues be an active area of research. It has been suggested that early positive response to anti-VEGF injections predicts outcome at three months ^46^. Conversely, recent OCTA data showed that deep capillary plexus damage predicts poor response to treatment ^47^. As with any invasive therapy, careful consideration of the risks and benefits of repeated intravitreal injections is necessary for each patient. These risks include serious ophthalmic complications such as endophthalmitis and retinal detachment as well as potential systemic complications such as hypertension, proteinuria, impaired wound healing, and increased risk of cardiovascular events ^48^.

## Update on dexamethasone and fluocinolone acetonide

Despite the dominance of anti-VEGF agents, there is still a role to play for intravitreal corticosteroids. Steroids work by inhibiting leukostasis, enhancing barrier function of tight junctions, and mitigating release of local inflammatory factors, including VEGF ^49^. The Macular Edema: Assessment of Implantable Dexamethasone in Diabetes (MEAD) study examined the effectiveness of a dexamethasone implant versus sham for the treatment of DME. The implant groups (0.7 and 0.35 mg) had higher percentages of patients achieving at least 15 ETDRS letters gained at 3-year follow-up compared with the sham group (22%, 18%, and 12%, respectively). A pooled analysis of the MEAD study showed that patients in either implant group had a 12-month delay in two-step DR progression (36 versus 24 months) compared with sham patients ^50^. As with most steroid treatments, development of cataract and intraocular pressure (IOP) elevation were the most common side effects.

Fluocinolone acetonide (Iluvien, Alimera Sciences, Alpharetta, GA, USA) is another corticosteroid implant that has treatment benefit for at least three years. The Fluocinolone Acetonide in Diabetic Macular Edema study evaluated low-dose (0.2 μg per day) and high-dose (0.5 μg per day) fluocinolone implants in patients with DME who had received at least one laser treatment. At 36 months, 33% and 31.9% of patients in the low-dose and high-dose groups, respectively, had an improvement of 15 ETDRS letters or more, compared with 21.4% of sham patients. Of patients with a history of DME at least three years, 34% and 28.8% of low- and high-dose patients, respectively, gained 15 ETDRS letters or more, compared with 13.4% of sham patients. Moreover, an at least two-step improvement in DRSS occurred in 13.7% of low-dose and 10.1% of high-dose patients compared with 8.9% of sham patients. As with other steroid therapies, side effects included cataract and IOP elevation. IOP elevation was severe enough to require glaucoma tube shunt placement in less than 5% of low-dose patients and 8% of high-dose patients ^51^.

The results of DRCR Protocol U will also soon be available. This study will assess the short-term effects of combination intravitreal dexamethasone and ranibizumab therapy in eyes with persistent DME despite prior anti-VEGF therapy compared with continued anti-VEGF therapy alone.

## Summary of pharmacologic agents

The continued takeaway of these recent studies on pharmacologic agents is that early treatment of DME in NPDR or PDR is vital to halt and sometimes reverse retinopathy. De-escalation of anti-VEGF treatment is sometimes possible without compromising initial gains, although this is more often the case when therapy has been initiated early. Corticosteroids are beginning to take a place as second-line therapy for patients unresponsive to anti-VEGF agents or focal laser or both. This is especially applicable for pseudophakic patients who have passed a steroid IOP challenge.

## Conclusions

Despite the remarkable gains we have made in the treatment of DR and its complications, the primary strategy against this disease should be the ardent prevention of diabetes whenever possible. If diabetes is already present, tight glycemic control is the most important modality to prevent or control retinopathy, and control of other systemic factors such as lipid levels and blood pressure also plays an important role. New diagnostic approaches and imaging modalities may aid in capturing referable retinopathy at earlier stages. Once retinopathy develops, early and regular treatment is crucial to prevent permanent vision loss. While there remains a place for laser and incisional surgery, the role of pharmaceutical agents has expanded in recent years. Anti-VEGF agents have become the first-line agents for the treatment of center-involved DME. In addition, intravitreal VEGF inhibitors have been proven to be effective in regressing DR. Inhibition of VEGF has resulted in an effective treatment to preserve vision in patients with DR. Future treatment strategies might focus on less invasive delivery methods or alternative therapeutic targets. In the early stages of DR, when neurodegeneration plays an important role, intravitreal anti-VEGF injections may represent an overly aggressive approach. In these situations, topically delivered therapies, such as endogenous neuroprotective substances, hold exciting promise ^52^. Along with the continued vigilance of clinicians, the expanding armamentarium of diagnostic and therapeutic options for DR will be an invaluable tool in the fight to reduce the impact of this devastating disease.
